# Prediction of protein solvent accessibility using PSO-SVR with multiple sequence-derived features and weighted sliding window scheme

**DOI:** 10.1186/s13040-014-0031-3

**Published:** 2015-01-31

**Authors:** Jian Zhang, Wenhan Chen, Pingping Sun, Xiaowei Zhao, Zhiqiang Ma

**Affiliations:** School of Computer Science and Information Technology, Northeast Normal University, Changchun, 1300117 P.R. China; School of Chemistry and Molecular Biosciences, The University of Queensland, Brisbane, Queensland Australia; The Engineering Laboratory for Drug-Gene and Protein Screening, Northeast Normal University, Changchun, 130117 P.R. China

**Keywords:** Solvent accessibility, Support vector regression, Protein sequence, Particle swarm optimization

## Abstract

**Background:**

The prediction of solvent accessibility could provide valuable clues for analyzing protein structure and functions, such as protein 3-Dimensional structure and B-cell epitope prediction. To fully decipher the protein-protein interaction process, an initial but crucial step is to calculate the protein solvent accessibility, especially when the tertiary structure of the protein is unknown. Although some efforts have been put into the protein solvent accessibility prediction, the performance of existing methods is far from satisfaction.

**Methods:**

In order to develop the high-accuracy model, we focus on some possible aspects concerning the prediction performance, including several sequence-derived features, a weighted sliding window scheme and the parameters optimization of machine learning approach. To address above issues, we take following strategies. Firstly, we explore various features which have been observed to be associated with the residue solvent accessibility. These discriminative features include protein evolutionary information, predicted protein secondary structure, native disorder, physicochemical propensities and several sequence-based structural descriptors of residues. Secondly, the different contributions of adjacent residues in sliding window are observed, thus a weighted sliding window scheme is proposed to differentiate the contributions of adjacent residues on the central residue. Thirdly, particle swarm optimization (PSO) is employed to search the global best parameters for the proposed predictor.

**Results:**

Evaluated by 3-fold cross-validation, our method achieves the mean absolute error (MAE) of 14.1% and the person correlation coefficient (PCC) of 0.75 for our new-compiled dataset. When compared with the state-of-the-art prediction models in the two benchmark datasets, our method demonstrates better performance. Experimental results demonstrate that our PSAP achieves high performances and outperforms many existing predictors. A web server called PSAP is built and freely available at http://59.73.198.144:8088/SolventAccessibility/.

**Electronic supplementary material:**

The online version of this article (doi:10.1186/s13040-014-0031-3) contains supplementary material, which is available to authorized users.

## Background

The solvent accessibility of a residue in a protein is a value that represents the solvent exposed surface area of this residue. It is crucial for understanding protein structure and function. As a result of the completion of whole-genome sequencing projects, the sequence-structure gap is rapidly increasing. Importantly, the knowledge of protein structures is a foundation for understanding the mechanism of diseases of living organisms and facilitating discovery of new drugs. The most reliable methods for identification of protein structure are X-ray crystallography techniques, but they are expensive and time-consuming. This leads to a central, yet unsolved study of protein structure prediction in bioinformatics, especially for sequences which do not have a significant sequence similarity with known structures [1]. To predict protein structure, the role of solvent accessibility has been extensively investigated as it is related to the spatial arrangement and packing of amino acids during the process of protein folding [2]. So it is often regarded as the first step in protein 3D structure prediction. As a measure of exposure to certain solvent, solvent accessibility can be used to identify what degree a residue is buried or exposed. Therefore, it also has important applications in predicting the active sites of a protein in protein-protein or protein-ligand interactions [3,4].

In earlier studies, prediction of solvent accessibility was regarded as a two-state (exposed or buried) or three-state (exposed, intermediate or buried) classification problem [5-10]. However, there is no generally accepted definition about the states of solvent accessibility. To meet the need of protein tertiary structure prediction, which requires a numerical measure of protein solvent accessibility, recent studies mainly focused on predicting the real values of the solvent accessibility.

In [11,12], Ahmad and Wang extracted and analyzed features from protein sequences combined with different statistical approaches. Results showed that these methods achieved a MAE of 18.5–19.7% on CB502 dataset. Shortly after that, Adamczak [13] made the first trial on position-specific scoring matrix (PSSM) profile, which was a 20 dimensional matrix that provides log-odds scores for finding a particular matching amino acid in the target sequence, to train an artificial neural network (ANN) for the prediction. The result revealed a performance with an MAE of 15.3-15.8% on PFAM database [14]. Subsequently, to make more exact prediction, many methods were built on PSSM features and features excavated from sequences. These methods included multiple linear regression [15], multiple sequence alignment [16], energy optimization [17], support vector regression [18,19], neural network [20,21], pace regression [22], agent-based system [23] and k-nearest neighbor (KNN) [24]. Among these methods, the lowest MAE achieved on CB502 dataset was approximately 14.8%, and the highest PCC was 0.68.

Although several methods were proposed for solvent accessibility prediction, the reported performance is far from satisfactory. There are some possible points concerning the performance: (i) it is well known that the protein sequences contain enormous amounts of information. However, the methods of feature extraction in most of these papers were based on a single technique; thus, it is inevitable that some useful information would be missed. In order to obtain more useful information, we explore various sequenced-derived features, which have been observed to be associated with the solvent accessibility or ever used in the similar tasks. The features used in this study consisted of PSSM profiles, protein secondary structure features (global and local information), sequence-based features (protein chain length and residue position), protein native disorder features and protein physicochemical features (hydrophilicity, flexibility, accessibility, polarity, exposed surface and turns). Experiments on our newly-compiled dataset show that the new introduced features can better describe the protein solvent accessibility; (ii) in protein, the adjacent residues always have an impact on the central target residue [18-24]. Almost all the studies treated the influence of each residue in the window equally. However, the residues in the sliding windows contribute differently on the central residue. In order to differentiate the various contributions, we proposed a weighted sliding window scheme; (iii) most machine learning tools are sensitive to the choice of parameter settings. Different parameters on the same machine learning algorithm could lead to varying results. Conventional parameter optimization for SVM is grid search. Grid search is a stiffly exhaustive searching approach which simply moves to a new parameters-node step by step independently. PSO is a robust optimization technique which has been successfully applied in many optimization problems. In PSO algorithm, more particles tend to converge into a good solution to search for better solutions; while grid-search algorithm simply moves to next node without considering previous performance. In this paper, instead of conventional grid-search, PSO is employed to search the global best parameters for the proposed predictor.

Based on above mentioned strategies, we propose an improved method for predicting protein solvent accessibility by using support vector regression (SVR) algorithm with multiple sequence-derived features, a weighted sliding window scheme and the PSO-based parameters optimization.

## Methods

### Datasets

To build the solvent accessibility database, we use PISCES culling server [25] with 25% sequence identity cutoff including X-ray structures (less than 3.0 Å resolutions and 0.3 of R-factor) which contain more than 100 residues and less than 1000 residues. As a result, 2312 protein chains with 816,621 residues (average length is 353) are collected to build the dataset PSAP2312 (May 2012).

In order to reach a consensus assessment with previous methods, two benchmark datasets, the Cuff & Barton dataset [5] and Manesh dataset [26], which were commonly used by previous methods to predict solvent accessibility are also used in this study. The Cuff & Barton dataset (CB502) includes 502 non-homologous protein chains with less than 25% pairwise-sequence similarity. The Manesh dataset (Manesh215) consists of 215 non-homologous protein chains with less than 25% pairwise-sequence similarity.

To test the stated-of-art web-servers used for practical application, we compile an independent dataset consists of 45 protein sequences with 11,750 residues (average length is 261) from PISCES culling server using the same filter scheme. None of these sequences occurs in the PSAP2312, CB502 and Manesh215 dataset. Hence, we can fairly compare the sequence-based solvent accessibility web prediction platforms.

All these datasets are available online at our PSAP web server.

### Feature encoding

The features used in this paper were encoded based on global and local information which are obtained from five sources: multiple alignment (PSIBLAST-based features), protein secondary structure (PSIPRED-based features), protein native disorder (DISOPRED-based features), protein primary structure information (sequence-based features), and residue physicochemical properties (physicochemical-property-based features).

#### PSI-BLAST-based features

Evolution is an eternal process which impenetrate the whole history of life [27,28]. Previous studies pointed out those differences in amino acid replacement dynamics associated with solvent accessibility status [29]. To generate evolutional profiles, multiple sequence alignments are preformed with default parameters (3 iterations and 0.001 of E-value cutoff) against the NCBI non-redundant protein sequence database, which has been filtered to remove the transmembrane regions, low-complexity regions and coiled-coil segments. PSI-BLAST [30] profile includes a 20×*L* PSSM [31], where *L* is the length of the protein chain, and each residue in the protein is encoded by an evolutionary information vector of 20 dimensions (Additional file 1). A sliding window of *N* neighboring residues is used to represent the evolutionary information of a sequence. The score values are normalized by standard logistic function:

1 $$ x\hbox{'}=\frac{1}{1+ \exp \left(-x\right)} $$

where *x* is the score derived from the PSSM profile and *x*’ is the standardized value of *x*.

An additional flag which indicates the C-terminal or the N-terminal of a sequence is usually treated as a terminal feature, which is set to 1 to indicate the two terminals or 0 otherwise. Thus, each residue is encoded by 20 features from PSSM and 1 feature from terminal flag, totally 21 features.

(N-1)/2 pseudo terminal residues are respectively added in the beginning and the end of each sequence. If the upstream or downstream for a target residue is less than 4, the lacking residues will be filled with dummy code *X*. For the pseudo terminal residue, the value of terminal flag feature is 1 and the value of evolutionary information features are 0. Finally, each protein residue is represented by (20+1) × *N* features. For instance, when the window is 9, we add 4 pseudo terminal residues in the front and the tail of the sequence respectively.

#### PSIPRED-based features

As the distributions of the residue depth values are different within three secondary structures this paper also includes secondary structure features [32]. PSIPRED applies two feed-forward neural networks to predict secondary structure using the results from PSI-BLAST [30]. The results of PSIPRED are encoded in terms of “C” for coil, “H” for helix, and “E” for strand. Local and global secondary structure features are derived from the outputs of the PSIPRED with default parameters. The local features are composed of 3×*N* features that concern probabilities in a window of *N* adjacent residues, where each residue is represented of C, H and E.

The global features are defined as follows:

2 $$ globalconten{t}_{\alpha }=\frac{conten{t}_{\alpha }}{ conten tH+ contentE+ contentC} $$

3 $$ globalsegmen{t}_{\alpha }=\frac{segmen{t}_{\alpha }}{ segmen tH+ segmentE+ segmentC} $$

where *α* = {*H*, *E*, *C*} is the percentage of secondary structures of type *α* in the sequence. *globalsegment*_*α*_ is the number of segments that only contain one type of consecutive secondary structures *α*. [32] indicated that one or two consecutive helical residue could not form a helix segment, so they are replaced by coils when calculating the frequency of secondary structure segments. As a result, 3×*N*+3+3 features are obtained from the PSIPRED’s output files.

#### Native disorder features

Natively disordered or unstructured regions are found to be associated with molecular assembly, protein translation, modification and molecular recognition [33-35].Previous studies indicate that disordered regions are strongly correlated with local solvent accessibility areas [36-38]. RSA values are often used to find the disordered regions [38]. In this study, DISOPRED [39] are used to output the predicted possibility of each residue being natively disordered or ordered. In this encoding scheme, a residue is encoded by a 3×*N*-dimensional vector.

#### Sequence-based features

Chakravarty [40] and Pintar [41] figured out the protein sequence length is correlated with both maximum and average ASA, which could be represented by a nearly linear function. As the size of protein sequence increases, the average solvent accessible surface of each residue is expected to decrease. Hence, to describe the effect brought by the length of protein sequence, the sequence length feature is used and normalized by dividing the sequence length by 1000.

Additionally, almost all the residues at the terminal are on surface or close to the protein surface. The feature about residue position is defined as follows:

4 $$ position=1-\frac{\left|i-\left(L+1\right)/2\right|}{L/2} $$

where *L* is the length of the protein sequence. This feature represents the distance between the *i*th residue and the terminal residue.

#### Protein physicochemical features

Earlier studies had shown that the hydrophobic interaction between residues played a key role in protein binding [42,43]. These residues tended to form small patches on the protein surface with polar and charged residues. Zhang [38] observed that the flexibility of a residue was strongly influenced by the solvent accessibility of the adjacent neighbors and mobile sections of a protein often had high solvent accessibility. Also, turns were valuable information as they strongly correlated with exposed surface area. Incorporating this information, Petersen [44] built a neural network predictor in the research of Beta-turns, which proved to be a valuable attempt. Therefore we adopted six physicochemical-property-based features, namely hydrophilicity, flexibility, accessibility, polarity, exposed surface and turns to predict solvent accessibility [45].

### Analysis of the least square linear regression models for the RSA values relation between central residue and adjacent residues

Previous works [18-24] simply used sliding window to represent the influence of adjacent residues have on the central one. However, this method assumes that each of the residues in the window contributes the central residue equally. In order to investigate the accurate influence, we use least square linear regression model to quantify the RSA values relationship between central residue and adjacent residues in different window size from 5 to 13 using the entire PSAP2312 dataset (Additional file 2). Finally, the 9-residue-length window is chosen and computed as follows:

$$ \begin{array}{l}RS{A}_i=0.04169RS{A_i}_{-4}+0.14068RS{A_i}_{-3}+0.267318RS{A_i}_{-2}+0.39247RS{A_i}_{-1}+\hfill \\ {}0.39149RS{A_i}_{+1}+0.262833RS{A_i}_{+2}+0.13861RS{A_i}_{+3}+0.04328RS{A_i}_{+4}-0.5863\hfill \end{array} $$

where *i* represents the *i*th residue in the protein sequence and RSA_*i*_ denotes RSA estimate for the *i*th residue.

This linear regression model shows that the weight values decrease linearly and symmetrically, with the linear distance from the central residue. All weights are positive, which means that the residues in sliding window have promoting effect on the central residue. So, instead of simple sliding window, we use the weighted sliding window scheme to differentiate the contributions of adjacent residues on the central one.

### Regression machines

**Figure 1:**
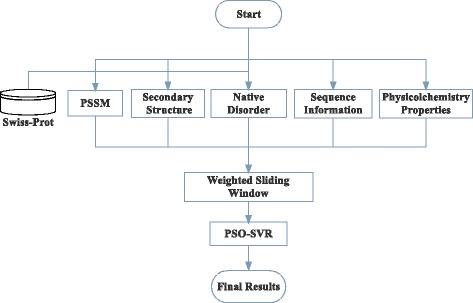
**The architecture of PSAP for protein solvent accessibility prediction.** Five different types of sequence-derived features are generated and constructed as input vector to build the PSO-SVR with weighted sliding window scheme.

In this paper, support vector regression (SVR) is expected to exhibit increased performance when compared with existing models. Figure 1 illustrates the architecture of our proposed model. SVR is to map the input features into a higher dimensional space using a kernel function to avoid optimization problem. The model produced by SVR depends only on a subset of the training data which lie on the margin. A regression package named LIBSVM (version 3.12) [46] is used in this study.

### Assessment of prediction accuracy

The performance of the proposed method is evaluated based on n-fold cross validation performed on PSAP2312, CB502 and Manesh215 datasets. The protein chains are randomly divided into n subsets to create cross validation folds. Here, we perform 3-fold cross-validation to maintain consistency with results reported in [15-19]. Furthermore, we also perform blind tests by building the prediction model on the PSAP2312, CB502 and Manesh215 and testing on the independent datasets.

Two widely used measurements for relative solvent accessibility (RSA) prediction are also adopted here to assess the performance of the proposed method: MAE and PCC [11-13,15-24], which are defined as follows:

5 $$ MAE=\frac{1}{N}{\displaystyle \sum_{i=1}^N\left|\frac{x_i-{y}_i}{x_i}\right|} $$

6 $$ PCC=\frac{{\displaystyle \sum_{i=1}^N\left({x}_i-\overline{x}\right)\left({y}_i-\overline{y}\right)}}{\sqrt{\left[{\displaystyle \sum_{i=1}^N{\left({x}_i-\overline{x}\right)}^2}\right]\left[{\displaystyle \sum_{i=1}^N{\left({y}_i-\overline{y}\right)}^2}\right]}} $$

where x_i_ and y_i_ are the real and predicted RSA values of the ith residue in the sequence respectively, while $\overline{x}$ and $\overline{y}$ are the corresponding mean values. *N* is the length of the protein sequence. MAE is used to quantitatively measure the deviation between the predicted and real values of relative solvent accessibility. PCC is employed to quantify the relationship between predicted and real values. The value of PCC is between −1 and 1. When the value of PCC is −1, {*x*_*i*_} and {*y*_*i*_} are fully negative correlation. On contrary, when the value of PCC is 1, {*x*_*i*_} and {*y*_*i*_} are fully positive correlation. The correlation between {*x*_*i*_} and {*y*_*i*_} is increased with increasing PCC value.

## Results and discussion

RSA is calculated by dividing the real ASA by the maximum solvent accessibility according to Ahmad’s work that uses extended tri-peptides (Ala-X-Ala) [47]. Therefore, to attain the RSA of a residue, ASA should be derived first. In this paper, we downloaded all the PDB files in PSAP2312 and CB502 and computed the accurate solvent-accessible surface area for each protein using the Dictionary of Protein Secondary Structure program (DSSP) [48]. For Manesh215, the values of ASA in Manesh215 dataset were obtained using the ASC program [49] with van der Waals radii given by Ooi et al. [50]. In this paper, we directly use the ready-processed Manesh215 dataset from [17].

### Features analysis and optimal feature set

Compared with the previous works, we introduce sequence-based, native disorder and protein physicochemical features in this study. Table 1 shows the predictive performance based on the 3-fold cross-validations SVR approach for different combinations of the five types of features on PSAP2312. The performance proves that the last prediction model is the best one, that is, all five types of features make contributions to the prediction of the protein solvent accessibility. The reasonably good performance of last prediction model implies that the comprehensive feature encoding can effectively find out the information of each residue.

**Table 1:** **Combination of different types of Sequence-derived features for SVR predictors on PSAP2312**

**Feature**	**PSAP2312**	
	**MAE (%)**	**PCC**
PSSM^1^	17.3	0.49
PSSM+PS^2^	16.2	0.55
PSSM+PS+ DO ^3^	15.5	0.61
PSSM+PS+ DO +SS^4^	15.2	0.65
PSSM+PS+ DO +SS+PC^5^	14.8	0.67

^1^Position specific scoring matrix; ^2^protein sequence information; ^3^Native disorder; ^4^Secondary Structure features; ^5^physicochemical propensities.

### Comparing SVR with weighted K-nearest neighbor and generalized boosted regression

In addition to the SVR, weighted K-Nearest Neighbor (wKNN) [51] and Generalized Boosting Regression (GBR) [52] are two popular machine learning methods in bioinformatics. For the purpose of comparison, wKNN and GBR are used to construct the prediction models (implemented by R software). All models are constructed by combining five sequence-derived features mentioned above using 3-fold cross-validation. As shown in Table 2, SVR yield better best results among three models. In addition, the parameter optimization of wKNN and GBR is extremely time-consuming. Since SVR demonstrates better performance and runs much faster than wKNN and GBR, SVR is chosen as regression engine in this work.

**Table 2:** **The performance of different machine learning methods using 3-fold cross-validation**

**Method**	**PSAP2312**	
	MAE (%)	PCC
wKNN^1^	14.9	0.63
GBR^2^	15.1	0.64
SVR	**14.8**	**0.67**

^1^weighted K-Nearest Neighbor, kernel = triangular, k = 19; ^2^Generalized Boosting Regression, distribution = Gaussian, n.trees = 1000, shrinkage = 0.05, interaction.depth = 3; best results are shown in bold.

### Comparing PSO with grid-Search in parameters optimization

The SVR algorithm is sensitive to the choice of parameter settings. If they are not set properly, the algorithm may have a substandard performance [53]. Suggesting a good setting is thus a crucial problem. Conventional parameters selection in SVR is grid-search, whose goal is to search the best optimum point with the least function value in the predefined multi-dimensional grid. This method is inefficient and non-intelligent. In this study, particle swarm optimization (PSO) was adopted to explore the best C, *γ* and *ɛ* for SVR predictor.

PSO is a meta-heuristic algorithm, inspired by the social behavior of bird flocking, originally developed by Eberhart and Kennedy in 1995 [54]. In the PSO algorithm, a bird in a flock is symbolically represented as a particle, which can be considered as a simple agent “flying” through a problem space. A particle’s location in the multi-dimensional problem space represents one solution for the problem. When a particle moves to a new location, a new problem solution is generated. This solution is evaluated by a pre-establish fitness function that provides a quantitative value of the solution’s utility.

Due to the large number of sequences of the PSAP2312, which imposes time consuming parameterization of SVR, we adopted a compromise calculation solution from [32]. A subset is constructed by randomly selecting 100 sequences from each fold from original dataset PSAP2312. This sub dataset, which is consisted of 300 chains, is referred to as PSAP300. The PSAP300 is used to parameterize PSO-SVR model. As a result, grid search scheme results in C = 0.01, *γ* = 0.0025 and *ɛ* = 0.05, while PSO-SVR approach gives *C* = 0.00762, *γ* = 0.00130, *ɛ* = 0.04129. Finally, the latter set of parameters is applied to build the proposed prediction model based on PSAP2312. The performance of different parameter optimization methods using 3-fold cross-validation is showed in Table 3.

**Table 3:** **Performance of different parameter optimization methods using 3-fold cross-validation**

**Method**	**PSAP300**		**PSAP2312**	
	**MAE (%)**	**PCC**	**MAE (%)**	**PCC**
SVR	19.6	0.60	14.8	0.67
SVR-grid search^1^	17.3	0.67	14.7	0.69
PSO-SVR^2^	**16.8**	**0.69**	**14.1**	**0.75**

^1^kernel = Gaussian, C = 0.01, *γ* = 0.0025, *ɛ* = 0.05; ^2^kernel = Gaussian, C = 0.00762, *γ* = 0.00130, *ɛ* = 0.04129; best results are shown in bold.

### Comparison of different regression predictors

The results from PSO-SVR on CB502 and Manesh215 are listed in Table 4 together with the results from four recently predictors. These methods include EO [17], SVR [18], Real-SPINE [20], pace regression [22] and NetSurfP [21]. The PSO-SVR method yields an MAE of 13.2%-14.0% and a PCC of 0.74-0.73 on CB502 and Manesh215 respectively, both of which are better than those of the compared predictors. The MAE value on CB502 is about 2%~5% lower than previous predictors.

**Table 4:** **Comparison with other reported methods**

**Method**	**CB502**		**Manesh215**	
	**MAE (%)**	**PCC**	**MAE (%)**	**PCC**
EO	-	0.49	-	0.52
SVR	14.8	0.68	14.2	0.69
Real-SPINE	14.5	0.68	13.8	0.70
PR	-	-	**13.2**	0.64
NetSurfP	14.3	0.71	13.6	0.70
PSO-SVR	**14.0**	**0.73**	**13.2**	**0.74**

Unreported results are denoted by “-”; best results are shown in bold.

### Comparison of different classification predictors

The predicted RSA values are also transformed into binary RSA states (exposed and buried) for comparison with conventional state RSA predictors. We adopt the standard approach in which the state is defined based on the predicted RSA values and a predefined threshold. For instance, a 5% threshold means that if one residue’s RSA value is no less than 5%, it is regarded as exposed residue, otherwise it is buried. The predictors for comparison are pace regression [22], agent-based system [23], two-stage SVR [19], SVR [18]. In order to reach a consensus assessment with previous studies, the results are revealed based on a test on the independent training set of 30 sequences from Manesh 215 dataset to predict the remaining 185 proteins of Manesh215 (Table 5). The proposed PSO-SVR predictor yields an accuracy rate >80% at 5-40%, >87% at 50-60% and >90% at 70-90% threshold respectively. These experimental results show that the present RSA predictor can exactly classify the buried or exposed state of residues.

**Table 5:** **Experimental comparison between the proposed predictor and other reported classification predictors**

**Method**	**Accuracy for two-states (buried vs. exposed) prediction (%)**										
	**5%**	**10%**	**20%**	**25%**	**30%**	**40%**	**50%**	**60%**	**70%**	**80%**	**90%**
PR	76.8	74.8	75.3	76.7	77.7	79.8	86.3	-	-	-	-
Agent-based	79.7	78.4	77.0	77.0	77.1	79.3	85.1	-	-	-	-
Two-stage SVR	81.1	78.7	77.6	77.3	-	-	79.5	84.3	89.9	**95.0**	97.5
SVR	80.9	80.1	78.7	-	-	-	80.8	85.3	**90.7**	**95.0**	**97.8**
PSO-SVR	**83.9**	**83.7**	**82.8**	**82.5**	**81.6**	**80.4**	**88.0**	**87.6**	90.2	**95.0**	**98.2**

Unreported results are denoted by “-”; best results are shown in bold.

### Comparison of different servers for the independent dataset

To our knowledge, there are some solvent accessibility prediction methods with publicly available web servers. These methods are RSARF [55], NetSurP [21], Real-SPINE 3.0 [20] and SANN [24]. Except RSARF, all methods are predicted the real solvent accessibility values. In this paper, we adopt the most recent methods NetSurP, Real-SPINE 3.0 and SANN as the benchmark methods for comparison, and the result are shown in Table 6. Here, we train our sequence-based models on PSAP2312 dataset, the CB502 dataset and the Manesh215 dataset respectively, and then use them to predict the independent dataset. Three models produce the mean MAE scores of 13.9%, 14.0% and 14.3% and the mean PCC scores of 0.73, 0.71 and 0.70. When compared with above-mentioned sequence-based servers, our model yields the best performance.

**Table 6:** **Experimental performance of different servers for the independent dataset**

**Method**	**Data for server construction**	**Server**	**Independent dataset**	
			**MAE (%)**	**PCC**
NN	513 proteins	NetSurfP	14.5	0.66
NN	2640 proteins	Real-SPINE 3.0	14.2	0.69
KNN	5717 proteins	SANN	14.3	0.69
PSO-SVR	PSAP2312	Our PSAP	13.9	0.73
	CB502	Our PSAP	14.0	0.71
	Manesh215	Our PSAP	14.3	0.70

### Residue-specific variation in prediction error

**Figure 2:**
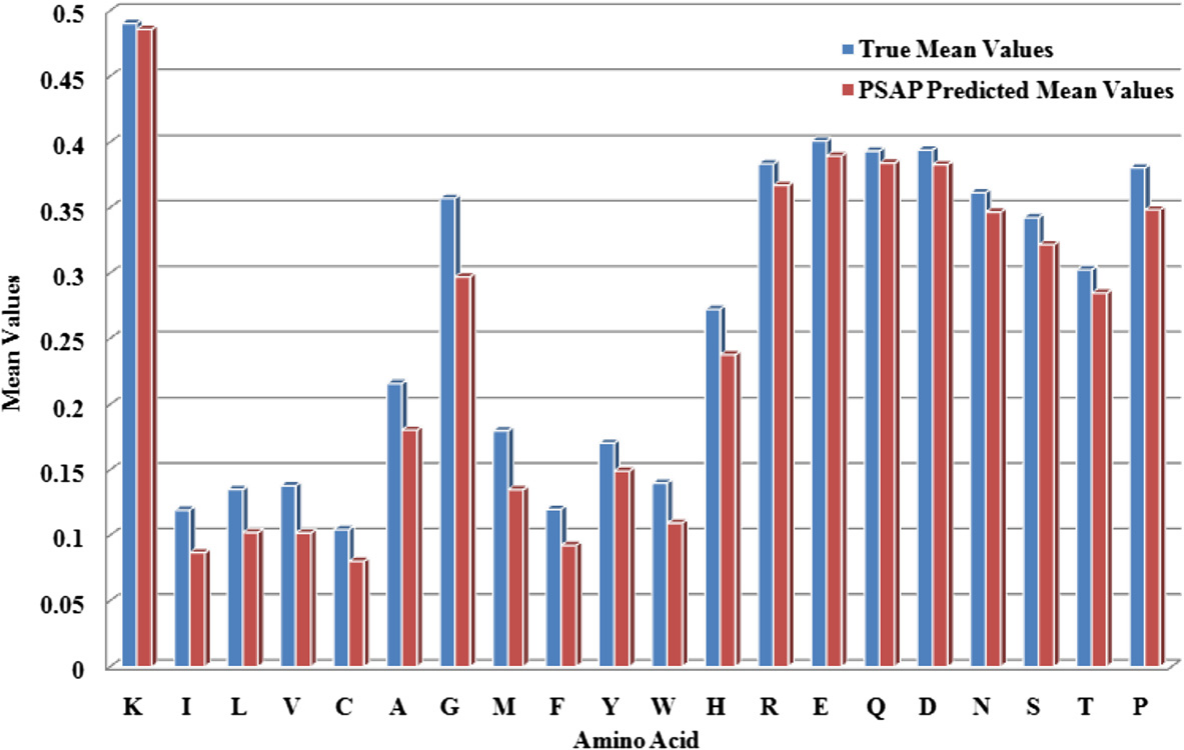
**True mean values and PSAP predicted mean values for 20 types of amino acid on PSAP2312 datasets.** The blue bar represents the true mean values, while the red bar represents the PSO-SVR predicted values.

**Figure 3:**
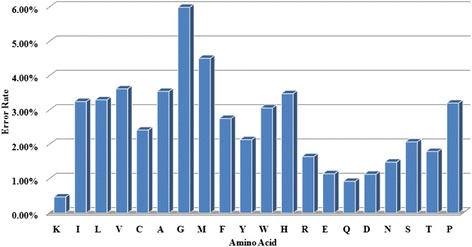
**20 types of amino acid mean predicted errors on PSAP2312 datasets.**

To discover the prediction performance of various types of residues, we further calculate the average RSA values in the PSAP2312 datasets for all 20 types of amino acids (Figures 2 and 3). In PSO-SVR predictor, 7 types of amino acid (K, R, E, Q, D, N, T) are predicted with <2% error. All types of amino acids are predicted with < 6% error in our method.

**Figure 4:**
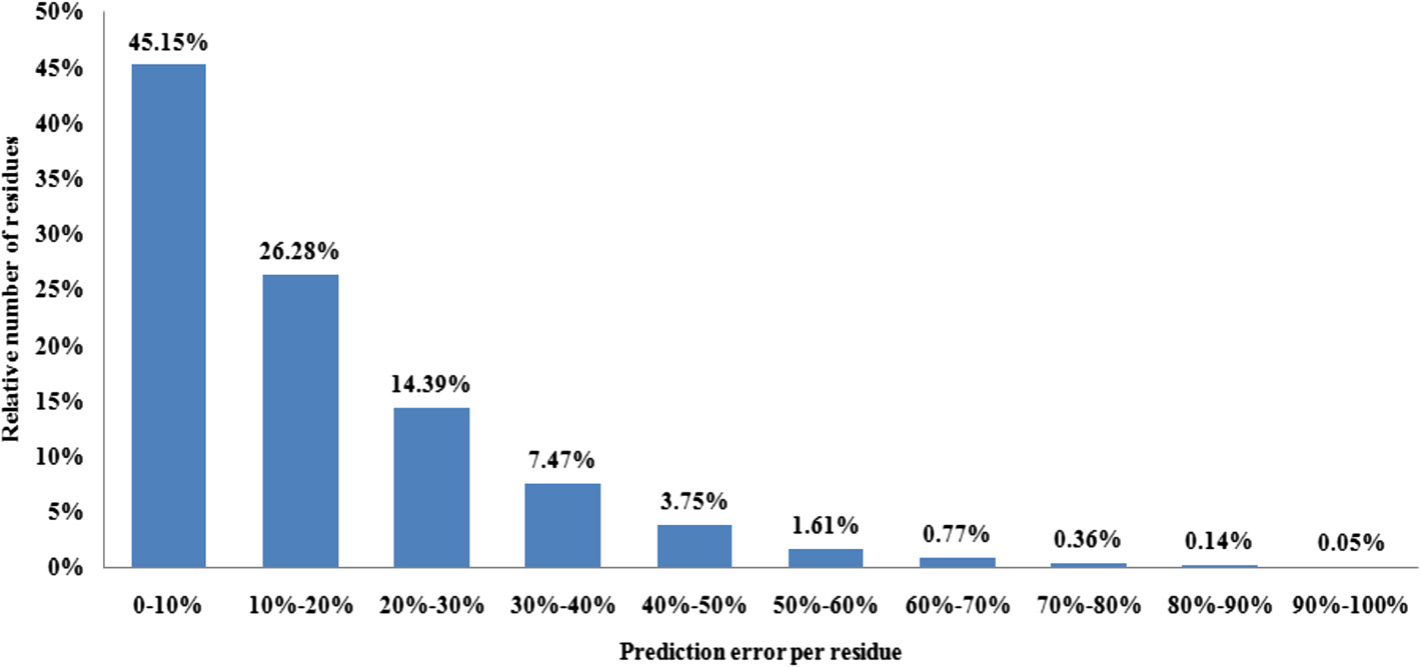
**Prediction error bar diagram showing the relative number of residues predicted within a given range of MAE on PSAP2312 dataset.**

In order to facilitate the comparison with previous studies, distribution of prediction error is calculated with respect to the variation of RSA values (Figure 4). More than 45% of all residues are predicted with less than 10% absolute error and less than 4% of all residues are predicted with greater than 40% error.

**Figure 5:**
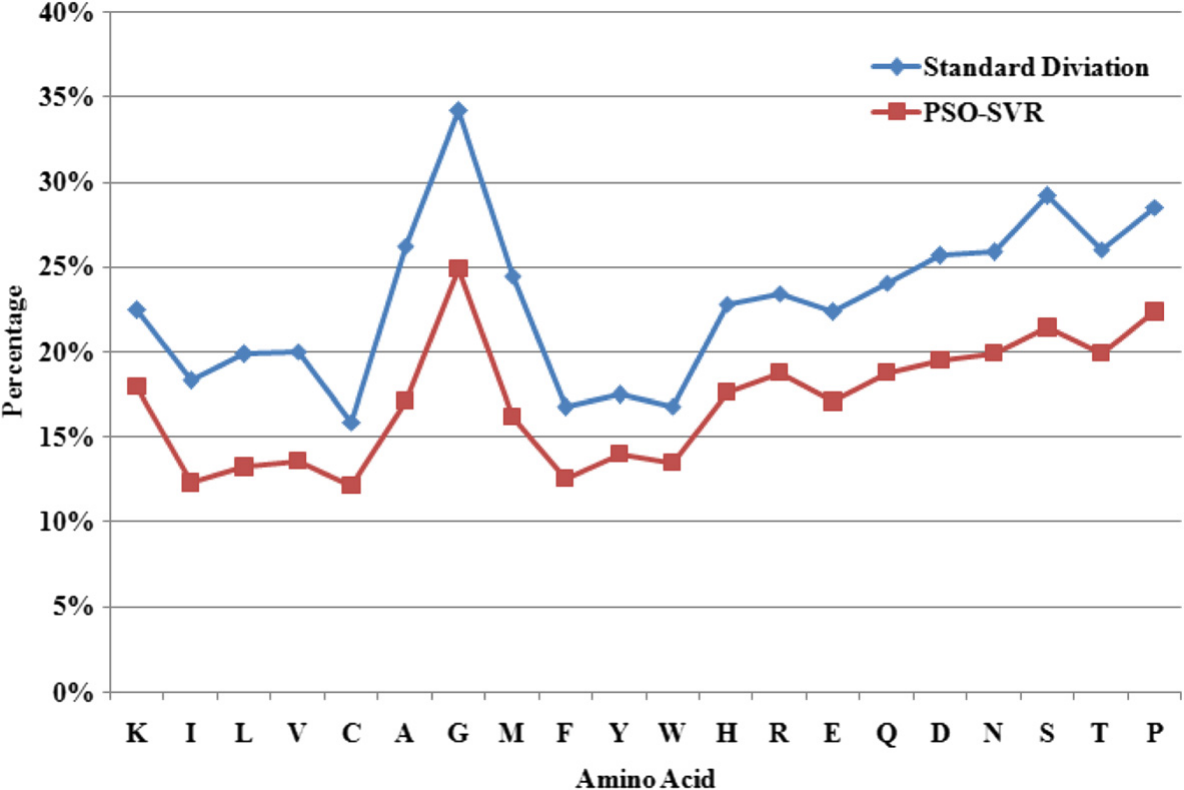
**Residue-specific prediction error and RSA variability.** Blue squares represent the prediction error of PSO-SVR approach on PSAP2312 dataset, while red circles represent standard deviation. The correlation between PSO-SVR approach and standard deviation is 96.9%.

What’s more, the prediction errors of 20 types of amino acids on PSAP2312 dataset are also calculated and showed on Figure 5. It also shows the variability of RSA in the overall dataset, which is represented by the standard deviation of the RSA values. The PSO-SVR method curve shows an excellent correlation at 96.9% with the standard deviation. The MAE values of PSO-SVR method for I, C, and F are less than 10%, which may due to the fact that the three types of residues are usually present in the interior of a protein (Figure 5 shows that the mean RSA values of I, C and F in the overall dataset are nearly 11.9%, 10.4% and 11.9% respectively).

## Conclusions

In this study, we present a new view to analyze the characteristics of solvent accessibility, and consider not only protein sequence information but also evolution similarity, secondary structure, native disorder and physicochemical properties of amino acids. A weighted sliding window scheme is proposed to differentiate the contributions of adjacent residues on the central one. PSO parameter optimization is adopted to search the global best C, *γ* and *ɛ* for SVR predictor. Experimental results on PSAP2312 and two benchmark datasets have demonstrated the efficacy of the proposed PSAP. The success of PSAP is due to several reasons include good benchmark datasets, sequence-derived features design, elaborate construction of the prediction model. Theoretically, accurate structure information could give the relatively accurate for the solvent accessibility area of a target residue. However, the number of proteins with completely structure information is far less than that with unknown structure information. As our method can predict the solvent accessibility from simple primary sequences in the absence of protein structures, it has more wide applications.

Generally, further improvements on the predictive performance rely on more discernable features or different combination of the currently proposed feature. To serve this purpose, more refined features could be generated from current features. In particular, the protein chain length and residue position features adopted in this work simply only reflects the linear relationship between mean solvent accessibility and the whole protein chain. However the relationship could be more complex and need more statistics. Moreover, the application of feature selection and other machine learning methods would be a future field that complements this study.

## Additional files

Additional file 1:
**PSI-BLAST-based Features.**
Additional file 2:
**Performance of Various window sizes for the least square linear regression model.**
