# Supplementary material for: Prediction of protein solvent accessibility using PSO-SVR with multiple sequence-derived features and weighted sliding window scheme
Source: BioData Min. 2015 Jan 31;8:3. doi: 10.1186/s13040-014-0031-3 (PMC4608127; doi:10.1186/s13040-014-0031-3)
Supplement: Additional file 1: — PSI-BLAST-based Features. [file 13040_2014_31_MOESM1_ESM.docx]

**Supporting Information S1**:

PSI-BLAST-based Features:

|  | **Number** | **Sequence** | **A** | **R** | **N** | **...** | **V** | **Terminal Flag** |
| --- | --- | --- | --- | --- | --- | --- | --- | --- |
|  | **Dummy Code** | ***X*** | **0** | **0** | **0** | **...** | **0** | **1** |
|  | **Dummy Code** | ***X*** | **0** | **0** | **0** | **...** | **0** | **1** |
|  | **Dummy Code** | ***X*** | **0** | **0** | **0** | **...** | **0** | **1** |
|  | **Dummy Code** | ***X*** | **0** | **0** | **0** | **...** | **0** | **1** |
|  | **1** | **K** | **-1** | **-2** | **0** | **...** | **-2** | **0** |
|  | **2** | **V** | **0** | **-3** | **-3** | **...** | **4** | **0** |
| **i-4** | **3** | **F** | **-2** | **-1** | **-3** | **...** | **-1** | **0** |
| **i-3** | **4** | **G** | **0** | **-1** | **4** | **...** | **-3** | **0** |
| **i-2** | **5** | **R** | **-1** | **3** | **0** | **...** | **-3** | **0** |
| **i-1** | **6** | **C** | **1** | **-2** | **-2** | **...** | **-1** | **0** |
| **i** | **7** | **E** | **-1** | **0** | **0** | **...** | **-2** | **0** |
| **i+1** | **8** | **L** | **-1** | **-3** | **-3** | **...** | **3** | **0** |
| **i+2** | **9** | **A** | **4** | **-2** | **-2** | **...** | **0** | **0** |
| **i+3** | **10** | **A** | **1** | **3** | **0** | **...** | **-2** | **0** |
| **i+4** | **11** | **A** | **3** | **-2** | **-2** | **...** | **-1** | **0** |
|  | **.** | **.** | **.** | **.** | **.** | **...** | **.** | **.** |
|  | **128** | **R** | **-1** | **-5** | **0** | **...** | **-2** | **0** |
|  | **129** | **L** | **-1** | **-2** | **-3** | **...** | **1** | **0** |
|  | **Dummy Code** | ***X*** | **0** | **0** | **0** | **...** | **0** | **1** |
|  | **Dummy Code** | ***X*** | **0** | **0** | **0** | **...** | **0** | **1** |
|  | **Dummy Code** | ***X*** | **0** | **0** | **0** | **...** | **0** | **1** |
|  | **Dummy Code** | ***X*** | **0** | **0** | **0** | **...** | **0** | **1** |

$$w=2\times4+1$$

An example of encoding a residue to its corresponding feature vector. In this example, a position of the protein sequence is represented by a 21-dimensional vector (20 amino acid values and a terminal flag).
