# Supplementary material for: Prediction of protein solvent accessibility using PSO-SVR with multiple sequence-derived features and weighted sliding window scheme
Source: BioData Min. 2015 Jan 31;8:3. doi: 10.1186/s13040-014-0031-3 (PMC4608127; doi:10.1186/s13040-014-0031-3)
Supplement: Additional file 2: — Performance of Various window sizes for the least square linear regression model. [file 13040_2014_31_MOESM2_ESM.docx]

**Supporting Information S2**:

^To quantify the relationship between the center residue and the adjacent residues in different window size, we use least square linear regression model and optimize the size of the local window based on 3-fold cross validation on the PSAP2312 dataset.^

^Table 1 and Figure 1 show that PCC display improvement with the increase of the window size of up to 9; while the MAE display decline under the same conditions. However, the further increase of the window size has little effect on the correlation. Thus, in this paper, we chose 9 as the window size to build the prediction model.^

^Section 1. Various window sizes for the least square linear regression model.^

^Section 2. Performance of Various window sizes for the least square linear regression model.^

^Section 1. Various window sizes for the least square linear regression model.^

***^Window size: 5^***

RSA*_i_*=0.2517RSA*_i-2_* + 0.6176RSA*_i-1_* + 0.637922RSA*_i+1_* + 0.27503RSA*_i+2_* - 0.7107

***^Window size: 7^***

RSA*_i_*= 0.08263RSA*_i-3_* + 0.166708RSA*_i-2_* + 0.478112RSA*_i-1_* + 0.496573RSA*_i+1_* + 0.18336RSA*_i+2_* + 0.09836RSA*_i+3_* - 0.6351

***^Window size: 9^***

RSA*_i_*=0.04169RSA*_i-4_* + 0.14068RSA*_i-3_* + 0.267318RSA*_i-2_* + 0.39247RSA*_i-1_* + 0.39149RSA*_i+1_* + 0.262833RSA*_i+2_* + 0.13861RSA*_i+3_* + 0.04328RSA*_i+4_* - 0.5863

***^Window size: 11^***

RSA*_i_*=0.03125RSA*_i-5_* + 0.06169RSA*_i-4_* + 0.11068RSA*_i-3_* + 0.190478RSA*_i-2_* + 0.37247RSA*_i-1_* + 0.38055RSA*_i+1_* + 0.19923RSA*_i+2_* + 0.12061RSA*_i+3_* + 0.06328RSA*_i+4_* + 0.03169RSA*_i+5_* - 0.4629

***^Window size: 13^***

RSA*_i_*=0.00622RSA*_i-6_* + 0.02154RSA*_i-5_* + 0.04173RSA*_i-4_* + 0.0765RSA*_i-3_* + 0.17316RSA*_i-2_* + 0.35247RSA*_i-1_* + 0.36138RSA*_i+1_* + 0.182833RSA*_i+2_* + 0.0852RSA*_i+3_* + 0.04246RSA*_i+4_* + 0.03149RSA*_i+5_* + 0.00721RSA*_i+6_* - 0.315

^Section 2. Performance of Various window sizes for the least square linear regression model.^

^Table I. Performance of Various window sizes.^

| Window size | MAE (%) | PCC (%) |
| --- | --- | --- |
| 5 | 8.8311 | 82.8153 |
| 7 | 7.9092 | 83.3318 |
| 9 | 7.3238 | 84.2569 |
| 11 | 7.3205 | 84.2573 |
| 13 | 7.3185 | 84.2604 |

^Figure I. Performance of Various window sizes.^

^
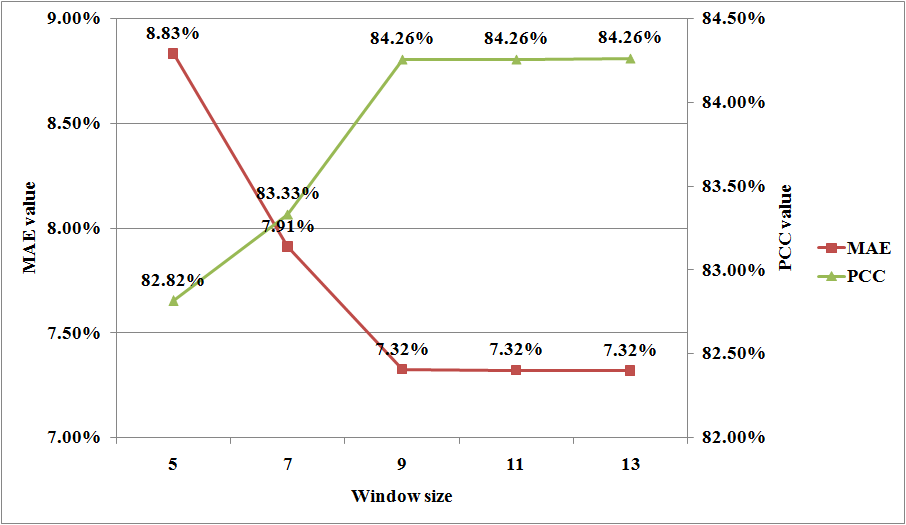
^
